# Covid-19 vaccines and thromboembolic complications

**DOI:** 10.1590/1677-5449.210167

**Published:** 2021-12-01

**Authors:** Marcone Lima Sobreira, Eduardo Ramacciotti, Adilson Ferraz Paschôa, Marcelo Fernando Matielo, Ivan Benaduce Casella, Guilherme Yazbek, Raphael de Athayde Soares, Bonno van Bellen, Marcos Arêas Marques

**Affiliations:** 1 Universidade Estadual Paulista “Júlio de Mesquita Filho” – UNESP, Botucatu, SP, Brasil.; 2 Universidade de Loyola, Chicago, Illinois, EUA.; 3 Universidade Estadual de Campinas – UNICAMP, Campinas, SP, Brasil.; 4 Universidade de São Paulo – USP, São Paulo, SP, Brasil.; 5 Hospital do Servidor Público Estadual de São Paulo, São Paulo, SP, Brasil.; 6 Real e Benemérita Associação Portuguesa de Beneficência de São Paulo, São Paulo, SP, Brasil.; 7 Universidade Federal do Estado do Rio de Janeiro – UNIRIO, Rio de Janeiro, RJ, Brasil.; 8 Universidade do Estado do Rio de Janeiro – UERJ, Rio de Janeiro, RJ, Brasil.

The current global pandemic caused by the novel coronavirus disease (Covid-19), which began in the city of Wuhan, in China, caused by a strain of the severe acute respiratory syndrome coronavirus 2 (SARS-CoV-2), infected around 18.77 million Brazilians, with approximately 525,500 deaths, according to data from the Ministry of Health, by July 2021.

With regard to its wide clinical spectrum, which varies from an asymptomatic form to severe acute respiratory syndrome (SARS), what has attracted the attention of angiologists and vascular surgeons are symptoms related to inflammation of the vascular system and hypercoagulability, leading to manifestations such as small vessel vasculitis and micro and macrovascular thrombosis of arteries and/or veins.^1^ A recent meta-analysis of 102 studies involving 64,503 patients infected by the SARS-CoV-2 virus reported that the frequency of venous thromboembolism (VTE) related to Covid-19 was 14.7% (95% confidence interval [95%CI]: 12.1% to 17.6%) and the frequency of arterial thromboembolism (ATE) was 4.0% (95%CI: 2.0% to 6.5%).^2^

The Covid-19 vaccines licensed for emergency use in Brazil in January 2021 demonstrated satisfactory safety and efficacy in clinical trials, and, despite the rapidity with which they were developed, all of them obligatorily followed rigid protocols and their data were evaluated by independent specialists and by Brazil’s National Agency for Sanitary Vigilance (Agência Nacional de Vigilância Sanitária - ANVISA). Vaccination eliminates or drastically reduces the risk of sickness or of severe forms of the disease.

The Covid-19 vaccines used in Brazil have the following efficacy rates:

- Fiocruz/Oxford University/AstraZeneca®: efficacy for moderate and severe cases (Brazil, United Kingdom, and South Africa): 70.42%;
- Instituto Butantan/Sinovac (CoronaVac®): efficacy for moderate and severe cases: 50.39%;
- Janssen Pharmaceuticals®/Johnson & Johnson® (Ad26.COV2. S): efficacy for moderate and severe cases: 66% (Latin America) and 72% (United States);
- Pfizer®/BioNTech® (Comirnaty®): efficacy for moderate and severe cases: 95%.

## VASCULAR MANIFESTATIONS OF COVID-19 VACCINES

### Arterial and venous thromboembolic events

Smadja et al.^3^ analyzed the occurrence of VTE and ATE in patients who had been vaccinated against Covid-19, using the VigiBase database (World Health Organization [WHO]), and reported that from December 13, 2020 to March 16, 2021 (94 days), there were 2,169 thrombotic events (795 venous and 1,374 arterial) among the 361,734,967 people who had been vaccinated, breaking down as 1,194 in people vaccinated with Pfizer®, 333 in those vaccinated with Moderna®, and 642 in people vaccinated with AstraZeneca®. The rate of notification of cases of VTE and ATE over that period for the total number of people vaccinated was 0.21 (95%CI: 0.19% to 0.22%) cases of thrombotic events per 1 million people vaccinated/day. For VTE and ATE, the rates were, respectively, 0.075 (95%CI: 0.07% to 0.08%) and 0.13 (95%CI: 0.12% to 0.14%) cases per 1 million people vaccinated/day. In Brazil, the rate was 0.89 events for each 100,000 doses administered, which is lower than the expected rate for the general population. As such, the risk-benefit profile of the vaccine is still favorable for these events.^4^

### Thrombosis with Thrombocytopenia Syndrome (TTS)

Rare cases of thrombocytopenia were also reported and, despite the favorable risk-benefit profile, some European countries decided to no longer recommend the AstraZeneca® vaccine for women younger than 55 or 60 years.^4^ The plausible explanation for the combination of VTE in atypical venous sites (cerebral and splanchnic veins) with thrombocytopenia is that an immune response is being triggered against platelet factor 4, leading to a major increase in activation and consumption of platelets, similar to heparin-induced thrombocytopenia in people who have not previously been exposed to heparins. D-dimer levels tend to be greatly elevated, but with normal fibrinogen levels.^5^^,^ 6 These thromboses occur around 4 to 24 days after administration of the vaccine^5^ and predominantly in women aged 20 to 50 years of age.^5^^,^ 6 Clinical status tends to be severe and there are reports of mortality exceeding 50%.^5^^,^ 6

Both the WHO and the European Medicines Agency published favorable opinions, stating that these events are extremely rare and that the benefits of the AstraZeneca® vaccine (reducing morbidity and mortality related to Covid-19) greatly outweigh its potential risks, reiterating the importance of vaccination. Moreover, administration of the vaccine was resumed in the European countries that had suspended it. It should be pointed out that thromboembolic events occur naturally and frequently in the general population, regardless of vaccination.

In general, the number of serious thrombotic events associated with the AstraZeneca® vaccine varied from 5.5 to 7.6 per million people vaccinated, illustrating the rarity of these events.^7^^,^ 8 Mathematical models estimate that the risk of thrombosis associated with this vaccine decreases as age increases, falling from an initial baseline of 1.9 per 100 thousand vaccinated people aged 20 to 29 years to 0.4 per 100 thousand vaccinated people over the age of 80.^9^

Up to April of 2021, 7.98 million doses of the Janssen® vaccine had been administered, with just 15 confirmed cases of TTS,^7^ with greatest incidence among female patients in the under-50 age group. To date, the Food and Drug Administration recommends continuing to administer the Janssen® vaccine to all age groups, irrespective of sex, with the observation that its information leaflet should mention the risk of TTS in female patients less than 50 years old.^10^

Although there are few reports of adverse events related to the Sputnik V® vaccine, which is not yet being used in Brazil, it employs similar technology to the AstraZeneca® and Janssen® vaccines, which are both associated with TTS, which increases the likelihood that it would have the same side effects.^11^ However, in a phase 3 trial with 21,977 randomized adults, just one patient developed VTE, probably related to a preexisting comorbidity.^12^ There were no reports of VTE or ATE in a real-life study of Sputnik V®.^13^

Finally, TTS should be suspected in patients with thromboembolic events associated with thrombocytopenia (<150,000/mm^3^) within 28 days of vaccination. Clinical management should avoid use of any type of heparin, preferring direct action oral anticoagulants or fondaparinux. In more severe cases, intravenous human immunoglobulin can be used. Corticosteroids should also be considered if administration of human immunoglobulin is delayed.^10^
