# Supplementary material for: Covid-19 vaccines and thromboembolic complications
Source: J Vasc Bras. 2021 Dec 1;20:e20210167. doi: 10.1590/1677-5449.210167 (PMC8668085; doi:10.1590/1677-5449.210167)
Supplement: Translation

# Translation: Vacinas para covid-19 e complicações tromboembólicas

**Authors:** Marcone Lima Sobreira, Eduardo Ramacciotti, Adilson Ferraz Paschôa, Marcelo Fernando Matielo, Ivan Benaduce Casella, Guilherme Yazbek, Raphael de Athayde Soares, Bonno van Bellen, Marcos Arêas Marques

**Affiliations:** 1 Universidade Estadual Paulista “Júlio de Mesquita Filho” – UNESP, Botucatu, SP, Brasil.; 2 Universidade de Loyola, Chicago, Illinois, EUA.; 3 Universidade Estadual de Campinas – UNICAMP, Campinas, SP, Brasil.; 4 Universidade de São Paulo – USP, São Paulo, SP, Brasil.; 5 Hospital do Servidor Público Estadual de São Paulo, São Paulo, SP, Brasil.; 6 Real e Benemérita Associação Portuguesa de Beneficência de São Paulo, São Paulo, SP, Brasil.; 7 Universidade Federal do Estado do Rio de Janeiro – UNIRIO, Rio de Janeiro, RJ, Brasil.; 8 Universidade do Estado do Rio de Janeiro – UERJ, Rio de Janeiro, RJ, Brasil.

A atual pandemia mundial causada pela doença do novo coronavírus (covid-19), iniciada na cidade de Wuhan, na China, e causada pela cepa do coronavírus 2 da síndrome respiratória aguda grave (SARS-CoV-2), infectou cerca de 18,77 milhões de brasileiros, com aproximadamente 525.500 mortes, segundo dados do Ministério da Saúde até julho de 2021.

Em que pese seu amplo espectro clínico, que varia desde a forma assintomática até a síndrome respiratória aguda grave (SARS), os sintomas relacionados à inflamação do sistema vascular e à hipercoagulabilidade que levam a manifestações como vasculite de pequenos vasos e trombose micro e macrovascular de artérias e/ou veias têm chamado atenção dos angiologistas e cirurgiões vasculares^1^. Em uma metanálise recente com 102 estudos envolvendo 64.503 pacientes infectados pelo vírus SARS-CoV-2, a frequência de tromboembolismo venoso (TEV) relacionado à covid-19 foi de 14,7% (intervalo de confiança de 95% [IC95%]: 12,1% a 17,6%) e a de tromboembolismo arterial (TEA) foi de 4,0% (IC95%: 2,0% a 6,5%)^2^.

As vacinas para covid-19 licenciadas para uso emergencial no Brasil em janeiro de 2021 demonstraram ter segurança e eficácia satisfatórias em estudos clínicos, e, apesar da rapidez nos seus desenvolvimentos, todas passaram obrigatoriamente por rígidos protocolos e tiveram seus dados avaliados por especialistas independentes e pela Agência Nacional de Vigilância Sanitária (Anvisa). A vacinação elimina ou reduz drasticamente o risco de adoecimento ou das formas graves da doença.

As vacinas contra a covid-19 utilizadas no Brasil apresentam as seguintes taxas de eficácia:

- Fiocruz/Universidade de Oxford/AstraZeneca®: eficácia para casos moderados e graves (Brasil, Reino Unido e África do Sul): 70,42%;
- Instituto Butantan/Sinovac (CoronaVac®): eficácia para casos moderados e graves: 50,39%;
- Janssen Pharmaceuticals®/Johnson & Johnson® (Ad26.COV2. S): eficácia para casos moderados e graves: 66% (América Latina) e 72% (Estados Unidos);
- Pfizer®/BioNTech® (Comirnaty®): eficácia para casos moderados e graves: 95%.

## MANIFESTAÇÕES VASCULARES DAS VACINAS CONTRA A COVID-19

### Eventos tromboembólicos arteriais e venosos

Smadja et al.^3^ avaliaram a ocorrência de TEV e TEA em pacientes submetidos a vacinação contra a covid-19, utilizando o banco de dados da VigiBase (Organização Mundial da Saúde [OMS]), e relataram que, no período de 13 de dezembro de 2020 a 16 de março de 2021 (94 dias), entre as 361.734.967 pessoas vacinadas, ocorreram 2.169 eventos trombóticos (795 venosos e 1.374 arteriais), sendo 1.194 com a Pfizer®, 333 com a Moderna® e 642 com a AstraZeneca®. A taxa de notificação de casos de TEV e TEA, durante o período, entre o número total de pessoas vacinadas foi de 0,21 (IC95%: 0,19% a 0,22%) casos de eventos trombóticos por 1 milhão de pessoas vacinadas/dia. Para TEV e TEA, as taxas foram respectivamente 0,075 (IC95%: 0,07% a 0,08%) e 0,13 (IC95%: 0,12% a 0,14%) casos por 1 milhão de pessoas vacinadas/dia. No Brasil, a taxa foi de 0,89 eventos para cada 100.000 doses aplicadas, inferior à taxa esperada para a população em geral. Portanto, o perfil de risco-benefício da vacina ainda é favorável para esses eventos^4^.

### Síndrome trombocitopênica trombótica

Raros casos de plaquetopenia também foram relatados, e, apesar do perfil de risco-benefício favorável, alguns países da Europa passaram a não recomendar a vacina AstraZeneca® para mulheres abaixo de 55 ou 60 anos^4^. A explicação plausível para a combinação de TEV em sítios venosos atípicos (veias cerebrais e esplâncnicas) e trombocitopenia seria o desencadeamento de uma resposta imunológica contra o fator 4 plaquetário, que levaria a um grande aumento na ativação e no consumo plaquetários, de forma semelhante à trombocitopenia induzida por heparina, em indivíduos não expostos previamente às heparinas. Os níveis de dímero-D costumam estar muito aumentados, porém com níveis de fibrinogênio normal^5^^,^ 6. Essas tromboses ocorreram cerca de 4 a 24 dias após a administração da vacina^5^ e de forma predominante no sexo feminino, entre 20 e 50 anos de idade^5^^,^ 6. O quadro clínico costuma ser grave, e há relatos de mortalidade superior a 50%^5^^,^ 6.

Tanto a OMS quanto a Agência Europeia de Medicamentos emitiram parecer favorável, informando que os eventos são extremamente raros e que os benefícios da vacina AstraZeneca®, reduzindo a morbimortalidade relacionada à covid-19, superam em muito os seus potenciais riscos, o que reitera a importância da vacinação. Ademais, seu uso foi retomado nos países europeus. É necessário esclarecer que eventos tromboembólicos ocorrem natural e frequentemente na população geral, independentemente de vacinação.

Em geral, o número de eventos trombóticos graves associados à vacina AstraZeneca® variou entre 5,5 a 7,6 por milhão de pessoas vacinadas, caracterizando a raridade do evento^7^^,^ 8. Modelos matemáticos estimaram que o risco de trombose associado a essa vacina decresceu com o aumento da faixa etária, sendo inicialmente de 1,9 para 100 mil vacinados entre 20 e 29 anos e caindo para 0,4 para 100 mil vacinados acima dos 80 anos^9^.

Até abril de 2021, 7,98 milhões de doses da vacina da Janssen® foram aplicadas, com apenas 15 casos confirmados de STT^7^, sendo a maior incidência em pacientes do sexo feminino, com faixa etária menor que 50 anos. A *Food and Drug Administration*, até o momento, recomenda a manutenção da vacina da Janssen® em todas as faixas etárias, independentemente do sexo, com a observação de que deva constar, na bula, o risco de STT em pacientes do sexo feminino com menos de 50 anos de idade^10^.

Apesar de poucos relatos de eventos adversos relacionado à vacina Sputnik V®, ainda não utilizada no Brasil, ela apresenta uma tecnologia semelhante às vacinas AstraZeneca® e Janssen®, ambas associadas a STT, o que aumenta a possibilidade de ter os mesmos efeitos colaterais^11^. Porém, em estudo de fase 3, entre 21.977 adultos randomizados, apenas um paciente desenvolveu TEV, provavelmente relacionado a comorbidade preexistente^12^. Não houve relatos de TEV ou TEA em estudo de vida real com a Sputnik V®^13^.

Por fim, deve-se suspeitar de STT em pacientes com eventos tromboembólicos associado a plaquetopenia (<150.000/mm^3^) dentro de 28 dias após a vacinação. No manejo clínico, deve-se evitar o uso de qualquer heparina, dando-se preferência aos anticoagulantes de ação direta de uso oral ou fondaparinux. Nos casos mais graves, pode-se utilizar imunoglobulina humana endovenosa. O uso de corticosteroides também deve ser aventado em caso de atraso no uso da imunoglobulina humana^10^.
